# Supplementary material for: A Comparison of Hypofractionated and Twice-Daily Thoracic Irradiation in Limited-Stage Small-Cell Lung Cancer: An Overlap-Weighted Analysis
Source: Cancers (Basel). 2021 Jun 9;13(12):2895. doi: 10.3390/cancers13122895 (PMC8229231; doi:10.3390/cancers13122895)
Supplement: Supplementary file 1 [file cancers-13-02895-s001.zip › cancers-1241802-supplementary.pdf]

Supplementary Materials

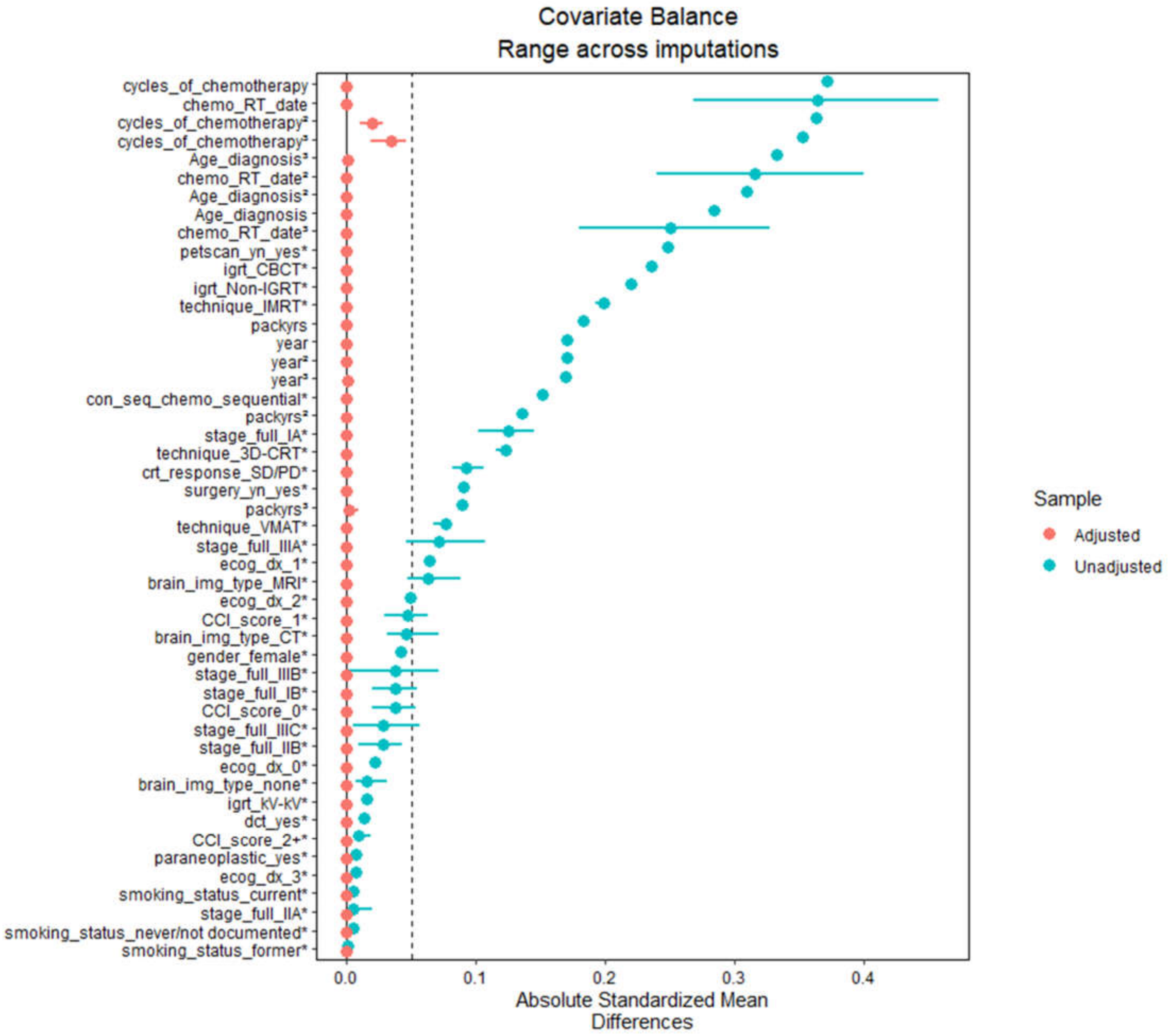

Figure S1. Love Plot with Standardized Mean Differences.

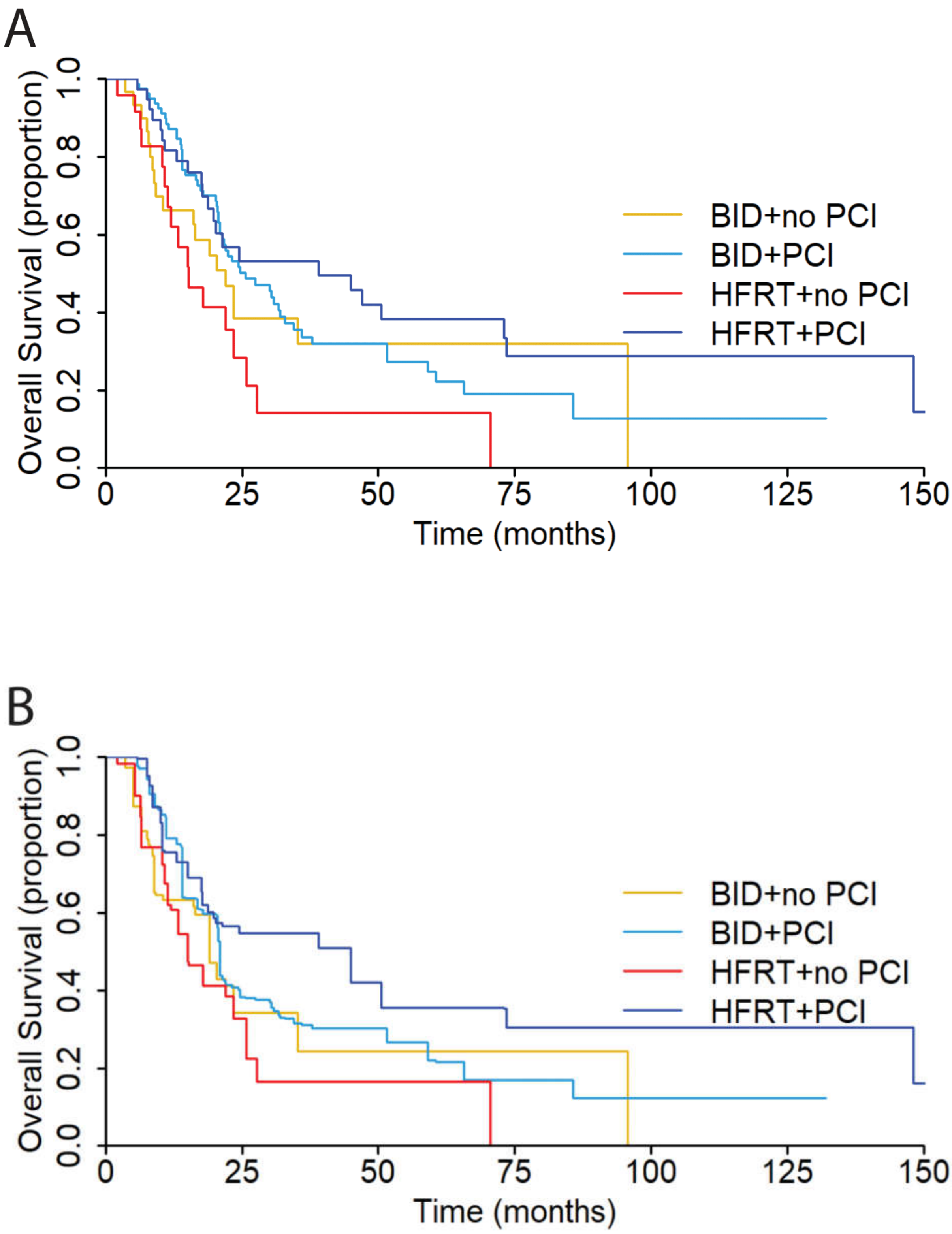

Figure S2. (A) Unweighted and (B) Overlap Weighted Kaplan Meier Estimates for Overall Survival Stratified by PCI and Radiotherapy Schedule. Abbreviations: BID – twice daily, HFRT – hypofractionated, PCI – prophylactic cranial irradiation.

Table S1. E-value for Multivariable Effect Estimates (HFRT versus BID [reference]).

Outcome	Unweighted		Overlap Weighted	
	**HR/OR**	**E-value (CI)**	**HR/OR**	**E-value (CI)**
OS	0.72	1.82 (1)	1.45	1.91 (1)
LRR risk	1.33	1.73 (1)	1.48	1.95 (1)
Thoracic Response	1.00	1.00 (1)	0.23	8.16 (1)
Any Grade 3+ Toxicity	1.31	1.55 (1)	1.67	1.91 (1)
Grade 3+ Esophagitis	1.16	1.37 (1)	1.14	1.34 (1)
Grade 3+ Pneumonitis	1.19	1.41 (1)	1.41	1.66 (1)

Abbreviations: OS – overall survival, LRR – locoregional recurrence, HR – hazard ratio, OR – odds ratio.
